# Full Coverage Hourly PM_2.5_ Concentrations’ Estimation Using Himawari-8 and MERRA-2 AODs in China

**DOI:** 10.3390/ijerph20021490

**Published:** 2023-01-13

**Authors:** Zhenghua Liu, Qijun Xiao, Rong Li

**Affiliations:** 1 Institute of Seismology, China Earthquake Administration, Wuhan 430071, China; 2 Key Laboratory of Earthquake Geodesy, China Earthquake Administration, Wuhan 430071, China; 3 Hubei Earthquake Administration, Wuhan 430071, China; 4 Faculty of Resources and Environmental Science, Hubei University, Wuhan 430062, China; 5 Hubei Key Laboratory of Regional Development and Environmental Response, Hubei University, Wuhan 430062, China

**Keywords:** PM_2.5_, Himawari-8, MERRA-2, random forest, gap-filled

## Abstract

(1) Background: Recognising the full spatial and temporal distribution of PM_2.5_ is important in order to understand the formation, evolution and impact of pollutants. The high temporal resolution satellite, Himawari-8, providing an hourly AOD dataset, has been used to predict real-time hourly PM_2.5_ concentrations in China in previous studies. However, the low observation frequency of the AOD due to long-term cloud/snow cover or high surface reflectance may produce high uncertainty in characterizing diurnal variation in PM_2.5_. (2) Methods: We fill the missing Himawari-8 AOD with MERRA-2 AOD, and drive the random forest model with the gap-filled AOD (AOD_H+M_) and Himawari-8 AOD (AOD_H_) to estimate hourly PM_2.5_ concentrations, respectively. Then we compare AOD_H+M_-derived PM_2.5_ with AOD_H_-derived PM_2.5_ in detail. (3) Results: Overall, the non-random missing information of the Himawari-8 AOD will bring large biases to the diurnal variations in regions with both a high polluted level and a low polluted level. (4) Conclusions: Filling the gap with the MERRA-2 AOD can provide reliable, full spatial and temporal PM_2.5_ predictions, and greatly reduce errors in PM_2.5_ estimation. This is very useful for dynamic monitoring of the evolution of PM_2.5_ in China.

## 1. Introduction

PM_2.5_ (particulate matter with an aerodynamic equivalent diameter less than or equal to 2.5 μm) emitted from anthropogenic and natural sources has a great adverse effect on human health, the climate and the environment [1,2,3]. China has suffered seriously from PM_2.5_ pollution in recent decades with rapid urbanization and industrialization [4,5,6]. Under the strategy of air pollution control, a ground-based observation network has been established to monitor air pollution in real time. Although it provides high-quality PM_2.5_ measurements every hour, there is a huge limitation in its spatial coverage due to the sparse and uneven distribution of monitoring stations. Therefore, satellite-based aerosol optical depth (AOD) has been widely used to estimate PM_2.5_ due to its strong relationship with ground-level PM_2.5_ [7,8,9]. Owing to the convenience of obtaining observations from monitoring stations in recent years, statistical approaches that combine satellite-retrieved AOD data with PM_2.5_ observations have become the main method to produce spatial continuous PM_2.5_ concentrations. These include the linear mixed effect (LME) model [10], generalized additive models (GAM) [11], geographically weighted regression (GWR)-related models [12,13] and hybrid models [10]. In essence, these statistical methods are still dominated by linear methods. With the development of deep learning, lots of novel machine learning methods such as random forest (RF), the deep neural network (DNN), the Extreme Gradient Boosting (XGBoost) and the Light Gradient Boosting Machine (LightGBM) have been induced to produce high accuracy PM_2.5_ concentrations over China [14,15,16].

In addition to the predicting model, the large rate of non-random missing information from AOD retrieval is an important factor that may lead to inevitably biases in monthly or yearly ground-level PM_2.5_ calculations [7,17,18]. Therefore, some researches tried to fill the gap in AOD retrieval to obtain full coverage ground-level PM_2.5_ concentrations. Xiao et al. combined Multi-Angle Implementation of Atmospheric Correction (MAIAC) with chemical transport model simulations through a multiple imputation method to fill the missing AOD [19]. Chen et al. developed a two-step interpolation method to replace the missing values in AOD [20]. Tuygun et al. merged MODIS, AERONET and MERRA-2 data to estimate PM_10_ concentrations.

These studies mainly paid attention to filling the gap of AOD products obtained from polar-orbiting satellites to estimate daily PM_2.5_ concentrations. The geostationary orbit satellite such as Himawari-8 equipped with the Advanced Himawari Imager (AHI) can provide a high temporal resolution AOD product that is useful for the diurnal variation investigation of air pollution. Recently, some researches have begun to estimate real-time hourly ground-level PM_2.5_ from the Himawari-8 AOD product [15,20,21,22]. However, most of these studies have focused on PM_2.5_ estimation models but not the essential relation between PM_2.5_ and AOD [23]. Xu et al. conducted a comprehensive investigation of the relationship between PM_2.5_ and the Himawari-8 AOD for the period of 2016–2018 in China, and found that due to different meteorological conditions, dominant aerosol type and AOD availability, the correlation between PM_2.5_ and the AOD fluctuate in different regions. Furthermore, cloud cover, land surface and the degree of pollution are different in major populated areas in China, such as Beijing–Tianjin–Hebei (BTH), the Pearl River Delta (PRD), the Yangtze River Delta (YRD) and Chengdu–Chongqing (CY). The error and missing rate in satellite-retrieved AODs due to these factors may introduce huge biases in hourly PM_2.5_ estimation and misunderstanding of the diurnal variation in PM_2.5_.

Focusing on the above issues, we fill the gaps of the Himawari-8 AOD with MERRA-2 AODs, and employ a RF model to estimate hourly ground-level PM_2.5_ in China from March 2017 to February 2018. After that, we present a comprehensive comparison of PM_2.5_ estimation based on the gap-filled AOD with that based on the non-gap-filled AOD, and then investigate the influence of the missing AOD on diurnal variation in densely populated regions including BTH, YRD, PRD and CY. This study provides full coverage of hourly PM_2.5_ predictions across China, which is helpful to obtain accurate diurnal variations in PM_2.5_ and reduce errors in exposure assessments to air pollution.

## 2. Materials and Methods

### 2.1. Data Source

#### 2.1.1. PM_2.5_ and AOD Data

The Environmental Protection Agency of China have published real-time hourly PM_2.5_ concentrations for the major cities in China on website (http://106.37.208.233:20035/) since January 2013. Ground-level hourly PM_2.5_ concentrations from 1441 monitoring stations in mainland China during March 2017 to February 2018 were collected from this website. The PM_2.5_ concentrations were measured by tapered element oscillating microbalance (TEOM) with the same ambient quality control standard.

The AHI sensor onboard Himawari-8 has provided hourly AOD (AOD_H_) at a 5 km resolution since 2014, and the coverage is 80° E–160° W and 60° N–60° S, which includes most regions of China. The validation of Himawari-8 AOD retrieval shows that Himawari-8 AOD has a high correlation with AERONET and Sun-Sky radiometer observation network over China [24]. We downloaded the L3 hourly AOD data from FTP address (ftp.ptree.jaxa.jp) provided by Japan Aerospace Exploration Agency (JAXA), and selected reliable AOD values through quality assurance flag that marked as ‘very good’ and ‘good’.

NASA’s Global Modeling and Assimilation Office (GMAO) produced the new atmospheric reanalysis product, namely, the Modern-Era Retrospective Analysis for Research and Applications, version 2 (MERRA-2) in 2017 [25]. MERRA-2 provides multi-decadal reanalysis of aerosol products that assimilate millions of aerosol observations from satellites and surface monitoring stations. Zhang et al. systematically evaluated the performance of Himawari-8 AODs and two reanalysis AOD datasets offered by MERRA-2 and Copernicus Atmosphere Monitoring Service (CAMS) over China [26]. They found that Himawari-8 and MERRA-2 AODs showed similar accuracies overall, and both presented significant diurnal variations. However, the accuracy of AOD products could be affected by pollution level, pollution distribution patterns and meteorological conditions. Recently, the accuracy of MERRA-2 AOD in China has been evaluated in many researches [27,28], and the results show that MERRA-2 AOD is in high agreement with both the Aerosol Robotic Network (AERONET) AOD and satellited-retrieved AOD. Studies have confirmed that the use of MERRA-2 AOD combined with machine learning models can estimate PM_2.5_ concentrations with reasonable accuracy [29]. Therefore, we selected MERRA-2 AODs to fill the gap of Himawari-8 AODs to obtain hourly full coverage AODs. The hourly MERRA-2 aerosol diagnostic dataset (AOD_M_) at the spatial resolution of 0.625° × 0.5° was used to fill gap of Himawari-8 AOD in this study.

#### 2.1.2. Auxiliary Data

The commonly used variables in previous researches including meteorological factors, normalized difference vegetation index (NDVI), population density, road network data, NO_2_ concentrations and DEM were selected as covariates in this study [30,31]. Meteorological data such as hourly air temperature, wind speed, specific humidity, surface pressure, total precipitation and boundary layer height were obtained from the Goddard Earth Observing System Assimilation System GEOS-5 Forward Processing (https://fluid.nccs.nasa.gov/weather/) at a spatial resolution of 0.25° × 0.3125°. NDVI data at 1 km spatial resolution was provided by MODIS 16-day global NDVI dataset (MOD13A2). The road length and density in a 5 km grid that represent vehicle emissions was calculated based on road network data from OpenStreetMap (https://openstreetmap.org/). The population distribution was collected from WorldPop at 1 km spatial resolution population density dataset (https://www.worldpop.org/). Integrated column concentrations of NO_2_ at a spatial resolution of 0.25° × 0.25° obtained from Ozone Monitoring Instrument (OMI) was used as a prediction variable as NO_2_ is an important precursor of PM_2.5_. The Shuttle Rader Topography Mission (SRTM) 90 m digital elevation model (DEM) product was used to characterize the influence of topography. All these datasets (Table 1) were resampled to the 5 km × 5 km Himawari-8 AOD grid covering the study region.

### 2.2. Model Development and Validation

**Figure 1:**
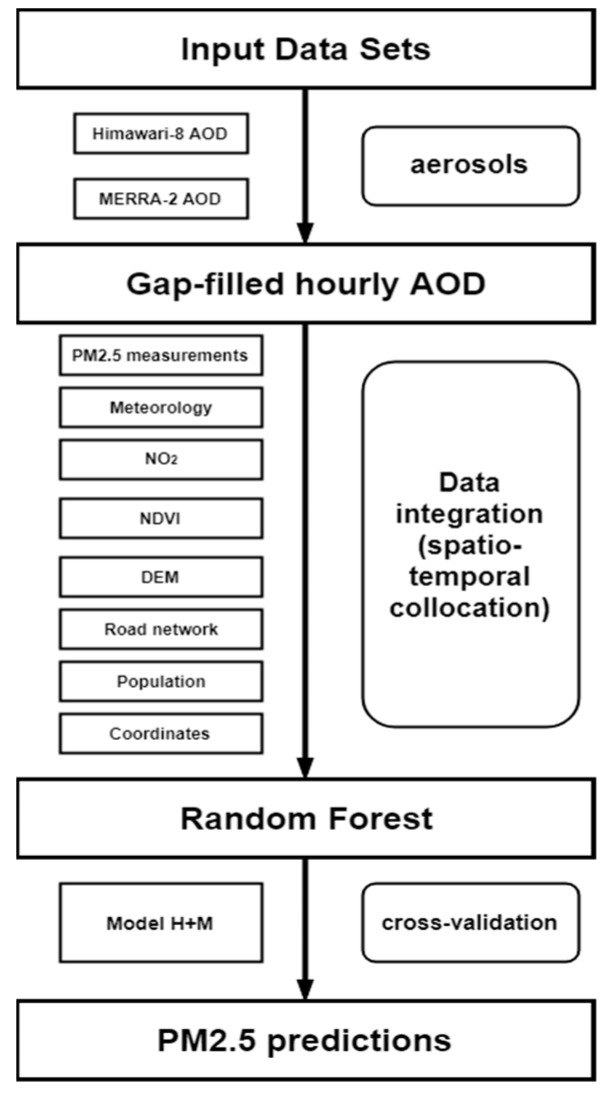
The workflow of PM_2.5_ estimation in this study.

The workflow of this study is shown in Figure 1. First, we resampled the AOD_M_ to the grid of AOD_H_, and used the resampled AOD_M_ to fill the missing AOD_H_. Second, the gap-filled AOD (AOD_H+M_) and other datasets including meteorology, NO_2_, NDVI, road network, population density and coordinates were integrated to the unified grid through spatial–temporal collocating. Finally, a random forest model derived by these predictor variables was used to predict ground PM_2.5_ concentrations.

Random forest model is an effective and relatively new machine learning method based on decision tree [32]. It is an ensemble of decision trees, and each sub-decision tree is constructed by sub-data drawn from a training set with replacement. The prediction results of random forests can be obtained by averaging the results of sub-decision trees. It is easy to evaluate the importance of each feature during the classification and reduce risk of overfitting. Compared with other deep learning algorithms, the random forest model is much simpler because there are only a few parameters needed to achieve optimal performance. Furthermore, the results can be more interpretable due to this model providing variable importance measures [33].

In this study, a random forest model was implemented by R package ‘Ranger’, a fast implementation of random forests. Two most important parameters in package ‘range’ are the number of trees (n_tree_) and the number of variables that can possibly be split in each node (m_try_). We set n_tree_ as 500 and m_try_ as 6 to obtain the balance of computing time and prediction accuracy after comparing results generated by different settings. Ten-fold cross-validation (CV) method based on all the data samples was used to ensure the robustness of the RF model in this study. Some stat metrics including the determination coefficient (R^2^), mean absolute error (MAE), relative prediction error (RPE), the root mean square error (RMSE) and index of agreement (IA) are often used to evaluate the accuracy of models [34,35]. We selected three commonly used statistical indicators (R^2^, RMSE, MAE) for PM_2.5_ estimation accuracy assessment. Here, we compared two RF models derived by AOD_H_ and the gap-filled AOD_H+M_ dataset to investigate the model performance after filling the gap of the Himawari-8 AOD with MERRA-2.

## 3. Results

### 3.1. Model Fitting and Validation

**Figure 2:**
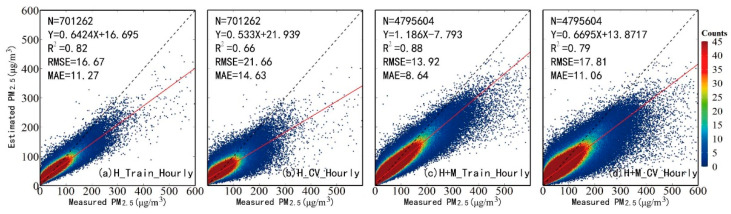
Density scatterplots of model fitting and 10-fold cross-validation for evaluating the model performance.

Figure 2 shows the scatterplots of the model fitting and cross-validation of hourly PM_2.5_, as produced by the RF models using AOD_H_ (a,b) and AOD_H+M_ (c,d), respectively. According to the model-fitting results, both RF models performed well with R^2^ values of 0.88 and 0.82, and RMSE values of 13.92 μg/m^3^ and 16.67 μg/m^3^, respectively. After performing cross-validation, the R^2^ values dropped slightly (0.79 and 0.66) and the RMSEs rose slightly (17.81 μg/m^3^ and 21.66 μg/m^3^). The results proved that the RF models work well in estimating PM_2.5_ concentrations with a little overfitting, whereas, after filling the gap of the Himawari-8 AOD with AOD_M_, the valid records of PM_2.5_-AOD rose from 701,262 to 4,795,604, and the accuracy of the model improved greatly. These results suggest that using AOD_H+M_ in the prediction model greatly reduces the uncertainty in the ground-level PM_2.5_ concentrations’ estimation overall.

**Figure 3:**
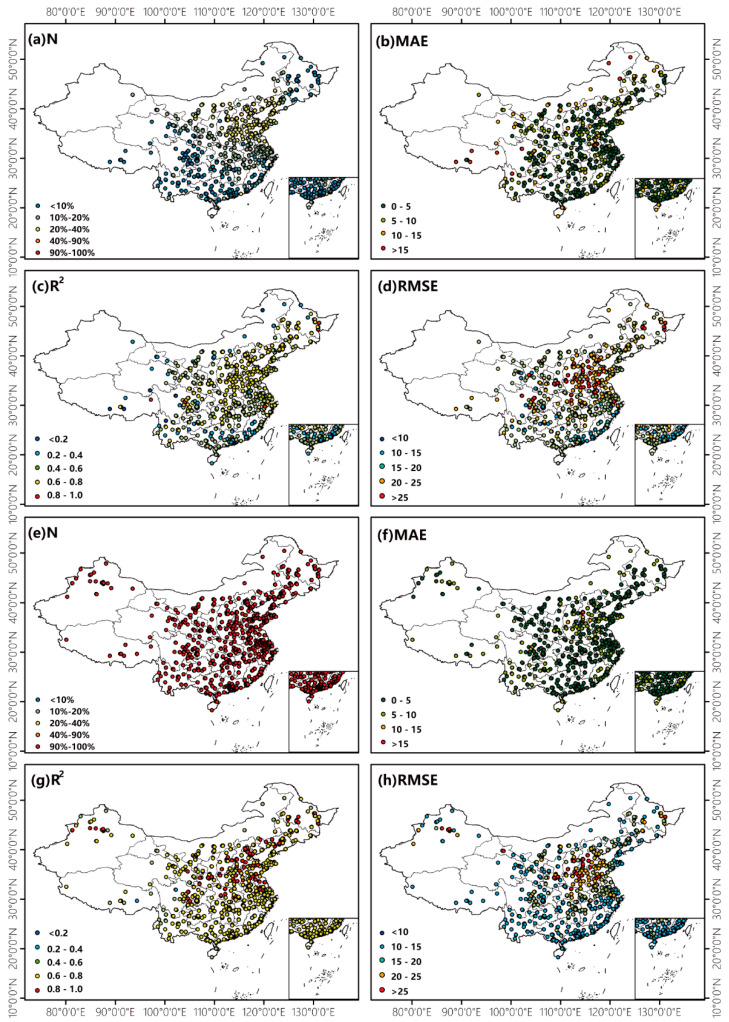
The valid observed frequency, CV RMSE, R^2^ and MAE of individual site across China produced by the RF model using AOD_H_ (**a**–**d**) and AOD_H+M_ (**e**–**h**), respectively.

To further investigate the performance of the models, the valid observed frequency (N), CV MAE, R^2^ and RMSE of individuals site across China were calculated and are shown in Figure 3. Figure 3a–d show the stat metrics of the AOD_H_-derived model, and Figure 3e–h show those of the AOD_H+M_-derived model. The observation frequency of hourly AOD_H_ varies greatly over regions due to the retrieval algorithm and cloud cover, and it is at its highest (~35%) in North China Plain, is lower (~16%) in central China and at its lowest in southwest China (~10%). After filling the gap of AOD_H_ with MERRA-2, the observation frequencies of most of the monitoring sites were up to 100%, except for a few sites where the measurements were missing. As the results show in Figure 3a–h, the accuracy of the AOD_H+M_-derived model improved significantly. At most of the monitoring stations across China, the site-specific CV R^2^ increased from ~0.6 to ~0.8, the RMSE decreased (5~10) μg/m^3^ and the values of MAE were below 5 μg/m^3^.

Although the overall accuracy of the model is reliable, the performance varies greatly in the typically populated regions. The values of CV R^2^ and RMSE are highest in the North China Plain, which has a dense network of monitoring sites and high PM_2.5_ concentrations. Before the AOD was gap filled with MERRA-2, the site-specific cross-validation accuracy was poor with an R^2^ value of 0.2~0.4 and RMSE value of 15~20 μg/m^3^ in southern China where it is constantly cloudy. However, the accuracy of estimation improved significantly with a CV R^2^ of 0.4~0.6 and a CV RMSE of 10~15 μg/m^3^ after the AOD_H_ gap was filled. The site-specific cross-validation result further suggests that using AOD_H+M_ in an RF model can improve estimation accuracy effectively, especially in long-term cloudy regions.

### 3.2. Performance of the Estimation Model on Temporal Scale

**Figure 4:**
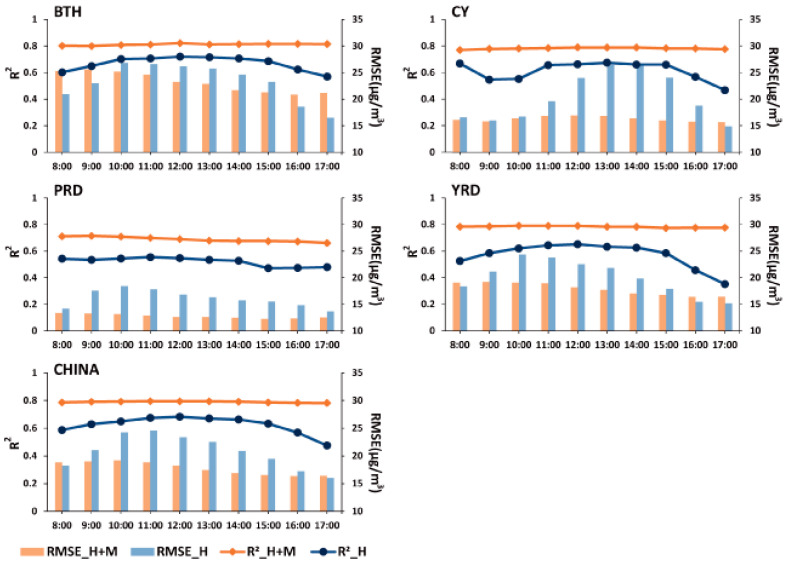
The temporal variation in CV R^2^ and RMSE values in hourly AOD_H_- and AOD_H+M_-derived PM_2.5_ over China and four typical regions.

The spatial variation in model performance is large due to the network density of monitoring sites, cloud cover, polluted degree, meteorological conditions, etc. How estimation errors vary over different timescales in different regions still requires further study. Figure 4 shows the temporal dependence of CV R^2^ and RMSE in hourly AOD_H_- and AOD_H+M_-derived PM_2.5_ (R^2^_H, RMSE_H, R^2^_H+M, RMSE_H+M) over China and four typical regions. The AOD_H_-derived model has reliable accuracy over China from 8:00~17:00 (CV R^2^_H: 0.6~0.7, RMSE_H: 15~25 μg/m^3^), and its performance varies over time obviously. As the observation frequency of AOD_H_ increases from 10:00~15:00, the overall CV R^2^_H improves and the CV RMSE_H increases.

The hourly error stat metrics (R^2^ and RMSE) of the AOD_H_-derived and AOD_H+M_-derived models varied greatly in different regions. Generally, the hourly CV R^2^ increased significantly and RMSE decreased over China after filling the gap of the Himawari-8 AOD with MERRA-2, but the degree of decline varied a lot at different times and in different spaces. As the most polluted region in China, BTH has high values of PM_2.5_ concentrations and a dense network of monitoring sites; the values of hourly CV R^2^ (R^2^_H is ~0.7, R^2^_H+M is ~0.8) and RMSE (RMSE_H and RMSE_H+M are both 15~25 μg/m^3^) are higher than other regions. The hourly RMSE values declined significantly (5–15 μg/m^3^) in PRD, CY and YRP where there is a high AOD missing rate (8~20%), but only declined a little in BTH (<5 μg/m^3^) from 10:00~15:00 after the AOD gap was filled. A previous study demonstrated that even though the PM_2.5_ model has a high accuracy of PM_2.5_ estimation overall, it performs relatively poorly in PRD, possibly due to the significant reduction in AOD observation caused by long-term cloud cover [21]. Our result suggests that using the MEERA-2 AOD to fill the gap of AOD_H_ can significantly reduce the uncertainty of PM_2.5_ estimation caused by the missing AOD. 

**Figure 5:**
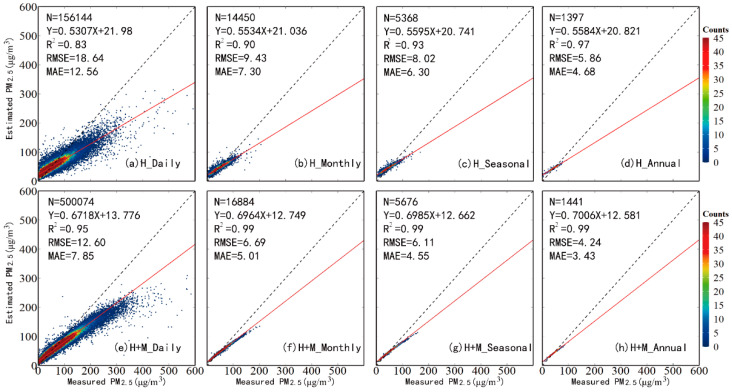
Density scatterplots of cross-validation result of hourly PM_2.5_ estimates derived by AODH and AODH+M, respectively, for (**a**,**e**) daily, (**b**,**f**) monthly, (**c**,**g**) seasonal, (**d**,**h**) annual.

To evaluate the model performance in different temporal scales, the cross-validated hourly PM_2.5_ estimated by AOD_H_ and AOD_H+M_ was used to predict daily, monthly, seasonal and annual average concentrations (Figure 5). The AOD_H+M_ model has very different R^2^ (0.95–0.99), RMSE (4.24–12.6 μg/m^3^) and MAE (3.43–7.85 μg/m^3^) values than the AOD_H_ model on four types of temporal scale (daily, monthly, seasonal and annual). The scatter plots of Figure 5a,e suggest that at a daily level, the accuracy of the AOD_H+M_-derived model (with values of R^2^, RMSE and MAE of 0.95, 12.6 μg/m^3^ and 7.85 μg/m^3^, respectively) is much higher than that of the AOD_H+M_-derived model (with values of R^2^, RMSE and MAE are 0.83, 18.64 μg/m^3^ and 12.56 μg/m^3^, respectively). These results indicate that the AOD_H+M_-derived PM_2.5_ model can capture PM_2.5_ variations more accurately on the daily scale. After averaging the hourly PM_2.5_ values to seasonal or annual averages, the differences in the statistical indicators (R^2^, RMSE and MAE) become smaller, as shown in Figure 5c,d,g,h.

## 4. Discussion

**Figure 6:**
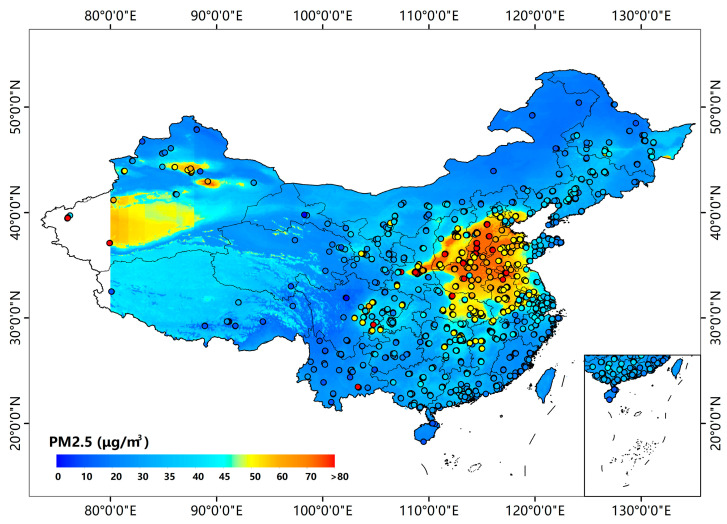
AOD_H+M_-derived annual PM_2.5_ concentrations and measurements of monitoring sites.

Full spatial–temporal coverage of PM_2.5_ concentrations can provide valuable information to interpret the formation, transport and removal process of pollutants. Usually, CV R^2^, RMSE and MAE values are used to assess the performance of a PM_2.5_ estimation model. Although most researches have similar R^2^ and RMSE values, they may differ significantly in the spatial and temporal distribution of PM_2.5_, which is also an important evaluation indicator of model performance. Figure 6 shows the spatial distributions of the AOD_H+M_-derived and ground-measured PM_2.5_ concentrations. The AOD_H+M_-derived PM_2.5_ concentrations agree well with ground-level observations, and the spatial patterns of the AOD_H+M_-derived PM_2.5_ concentrations are similar to the results reported in previous studies, especially in the typical regions such as BTH, YRD, PRD and CY [9,12]. The values of MAE are below 5 μg/m^3^ over most monitoring stations across China. The most polluted regions in China are Hebei, Shanxi, Henan and Shandong with annual mean PM_2.5_ concentrations of 70~80 μg/m^3^. Moreover, PM_2.5_ estimations in these places are highly biased, roughly in the range of 5–10 μg/m^3^. Under the long-term strict emission reduction policies from 2012, the PM_2.5_ concentrations have declined a lot in north China. The PM_2.5_ concentrations are distributed at ~40 μg/m^3^ in Xizang, and there may be high uncertainty due to the sparse distribution of the monitoring sites.

**Figure 7:**
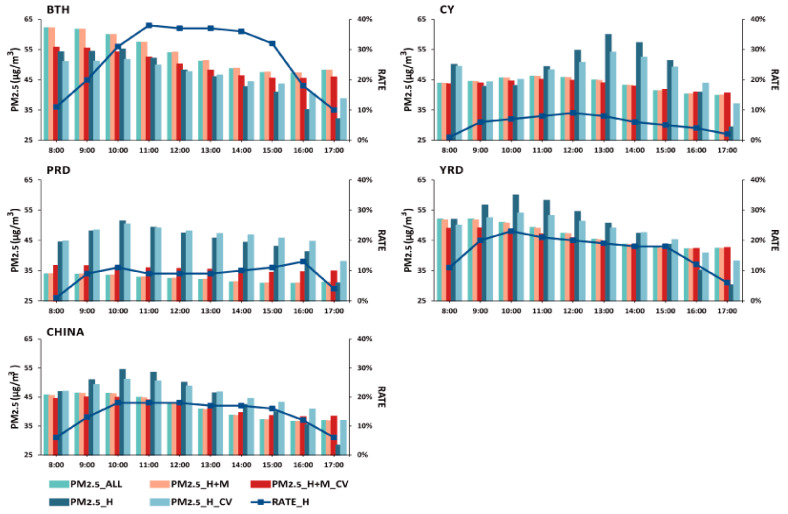
Time series of PM_2.5_ concentrations of all monitoring sites (PM_ALL), PM_2.5_ observations matched with AOD_H_ (PM_2.5__H), AOD_H+M_ (PM_2.5__H+M), AOD_H_-derived (PM_2.5__H_CV), AOD_H+M_-derived PM_2.5_ (PM_2.5__H+M_CV) and observation rates of Himawari-8 in China and four typical regions.

Some studies have suggested that the non-random missing AOD data may result in serious underestimation of the annual PM_2.5_ in BTH [7,19]. However, the influence of the observation frequencies in regions with relatively lower PM_2.5_ concentrations, such as PRD, CY and YRD, has been paid little attention. To further investigate the influence of the missing rate of the Himawari-8 AOD on PM_2.5_ diurnal variation, we first calculated the hourly observations from 8:00–17:00 of all monitoring sites (PM_2.5__ALL), then calculated the hourly PM_2.5_ observations matched with AOD_H_ (PM_2.5__H) and AOD_H+M_ (PM_2.5__H+M). We compared the average hourly PM_2.5_ derived by AOD_H_ (PM_2.5__H_CV) and AOD_H+M_ (PM_2.5__H+M_CV) with PM_2.5__ALL, PM_2.5__H and PM_2.5__H+M over the whole of China and four the typical regions shown in Figure 7. PM_2.5__H is higher than PM_2.5__ALL by about (5~10) μg/m^3^ from 9:00 to 16:00 across China, and this indicates that the model may overestimate the annual PM_2.5_ concentrations over China due to the missing AOD. These biases vary considerably in different regions due to their different pollution backgrounds. The average hourly PM_2.5_ concentration is underestimated in BTH because AOD is missing in some heavily polluted scenarios, while in less polluted regions such as PRD, CY and YRD, it is overestimated due to many clean scenarios being excluded when there is cloud cover. These biases may lead to huge misunderstandings of the diurnal variations in PM_2.5_ in China. After filling the gap of Himawari-8 AOD with MERRA-2 AOD, the values of hourly PM_2.5__H+M_CV approached the true diurnal variations that were better than the PM_2.5__H_CV values.

## 5. Conclusions

PM_2.5_ has a great influence on the atmospheric environment and health in China. Knowing the diurnal variation is important to understand the formation and evolution mechanism of PM_2.5_. Many studies have used hourly AOD products of geostationary satellites, including Himawari-8/AHI, to estimate hourly PM_2.5_ concentrations. However, due to cloud cover and surface characteristics, the non-random missing of Himawari-8 AODs may lead to misunderstandings and errors. In this study, the MERRA-2 AOD dataset is used to fill the gaps of the Himawari-8 AOD. Then, based on the gap-filled AOD and other auxiliary data, the RF model is used to predict hourly ground-level PM_2.5_ concentrations. PM_2.5_ concentrations derived from AOD_H+M_ and AOD_H_ are compared using different spatial and temporal scales. The impact of missing AOD on ground PM_2.5_ daily variation varies hugely in different regions. Annual hourly PM_2.5_ concentrations derived from AOD_H_ in the daytime (8:00–19:00) are lower than observations in heavily polluted BTH and much higher than those of less polluted regions such as PRD, CY and YRD. However, by filling the gaps of the Himawari-8 AOD with the MERRA-2 data, the accuracy of the estimating model and the ability to estimate diurnal variations of ground PM_2.5_ are greatly improved. This is very useful for dynamic monitoring of the evolution of PM_2.5_ in China.

## Figures and Tables

**Table 1:** Details of the data source used in this study.

Dataset	Variable	Content	Spatial	Temporal	Data Source
PM_2.5_	PM_2.5_	Particulate matter	In situ	Hourly	China Meteorological Administration
AOD	AOD_H_	Himawari-8 AOD	5 km × 5 km	Hourly	Himawari-8
	AOD_M_	MERRA-2 AOD	0.5° × 0.625°	Hourly	MERRA-2
Meteorology	PS	surface_pressure	0.25° × 0.3125°	Hourly	GEOS-5
	PBLTOB	pbltop_pressure			
	TS	surface_skin_temperature			
	T2M	2-meter_air_temperature			
	U2M	2-meter_eastward_wind			
	V2M	2-meter_northward_wind			
	TQL	total_precipitable_liquid_water			
	QV2M	2-meter_specific_humidity			
NO_2_ concentrations	NO_2_	Nitrate dioxide	0.25° × 0.25°	Monthly	OMIO2d
Land cover	NDVI	NDVI	1 km × 1 km	16 day	MOD13A2
Topography	DEM	Surface elevation	90 m	—	SRTM
Road network	RRDENSITY	Railways and roads density	5 km × 5 km	Yearly	OSM
	RRLENGTH	Railways and roads length		Yearly	
Population	POP	Population density	1 km × 1 km	Yearly	WorldPOP
Coordinates	LON	Longitude	—	—	
	LAT	Latitude	—	—	

## Data Availability

The Environmental Protection Agency of China has published real-time hourly PM_2.5_ concentration for the major cities in China on website (http://106.37.208.233:20035/) since January 2013. The L3 hourly AOD data from FTP address (ftp.ptree.jaxa.jp) is provided by Japan Aerospace Exploration Agency (JAXA). Meteorological data such as hourly air temperature, wind speed, specific humidity, surface pressure, total precipitation and boundary layer height are obtained from the Goddard Earth Observing System Assimilation System GEOS-5 Forward Processing (https://fluid.nccs.nasa.gov/weather/) at a spatial resolution of 0.25° × 0.3125°. NDVI data at 1 km spatial resolution is provided by the MODIS 16-day global NDVI dataset (MOD13A2). The road length and density in a 5 km grid that represent vehicle emissions is calculated based on road network data from OpenStreetMap (https://openstreetmap.org/). The population distribution is collected from WorldPop at 1 km spatial resolution population density dataset (https://www.worldpop.org/).
